# Serological and Immunological Markers in Dengue Fever: A Retrospective ELISA-Based Pilot Study from Trinidad and Tobago

**DOI:** 10.3390/diagnostics16152434

**Published:** 2026-08-01

**Authors:** Angel Justiz-Vaillant, Sachin Soodeen

**Affiliations:** Department of Pathology/Microbiology and Pharmacology, Faculty of Medical Sciences, University of the West Indies, St. Augustine 330912, Trinidad and Tobago; sachin.soodeen@my.uwi.edu

**Keywords:** dengue fever, ELISA, IgM, seroprevalence, flavivirus, Trinidad and Tobago

## Abstract

**Background**: Dengue fever remains a major mosquito-borne viral disease in tropical and subtropical regions and continues to represent an important public health challenge in the Caribbean. Despite endemic transmission in Trinidad and Tobago, limited laboratory-based studies have evaluated serological markers associated with recent dengue infection. **Objective**: To evaluate serological and immunological markers associated with recent dengue virus infection through the detection of anti-dengue virus immunoglobulin M (IgM) antibodies in serum samples obtained from Trinidad and Tobago using an enzyme-linked immunosorbent assay (ELISA). **Methods**: Anti-dengue virus IgM antibodies were detected using a commercial enzyme-linked immunosorbent assay (ELISA). Results were classified as positive, borderline or negative according to manufacturer-recommended criteria. Descriptive statistical analysis was performed to determine seroprevalence in anonymized serum samples. **Results**: A total of 161 serum samples were analyzed. Twenty samples (12.4%) demonstrated positive anti-dengue IgM reactivity, while 29 samples (18.0%) showed borderline reactivity and 112 samples (69.6%) were negative. Under the conservative analytical approach, the estimated dengue IgM seroprevalence was 12.4% (20/161; 95% CI: 7.7–18.6%). **Conclusions**: The estimated seroprevalence of dengue IgM in this pilot study was highly dependent on the analytical treatment of borderline ELISA results. Conservative classification provided the most cautious and reproducible estimate of recent dengue infection, while liberal interpretation substantially increased the apparent disease burden. Early viral replication triggers inflammation that progresses to cytokine-driven vascular damage, while cross-reactive humoral responses and host factors ultimately shape dengue severity. Ultimately, understanding the drivers of IgM cross-reactivity is essential for accurate dengue surveillance, outbreak detection, and clinical decision-making.

## 1. Introduction

Dengue fever remains one of the most important mosquito-borne viral diseases worldwide and continues to pose a major public health challenge in tropical and subtropical regions. The disease is caused by four antigenically distinct dengue virus (DENV) serotypes (DENV-1 to DENV-4), transmitted primarily by *Aedes aegypti* mosquitoes. An estimated 100–400 million dengue infections occur annually, placing nearly half of the global population at risk of infection [1].

The Caribbean remains an important endemic region for dengue virus transmission, with recurrent outbreaks influenced by climatic conditions, seasonal rainfall, vector ecology, and population immunity [2,3,4]. Trinidad and Tobago has experienced repeated dengue outbreaks over several decades, with evidence of circulation of multiple dengue virus serotypes [3,5]. Unfortunately, there are very few published serological studies from Trinidad and Tobago reporting dengue IgM seroprevalence. Most local publications focus on outbreaks, vector surveillance, or IgG seroprevalence rather than recent IgM infection. Despite the continuing public health importance of dengue in the country, relatively few laboratory-based studies have examined recent serological evidence of infection within the local population, limiting the availability of contemporary data to support surveillance and public health planning.

Laboratory diagnosis plays a central role in dengue surveillance because clinical manifestations are often non-specific and overlap with those of other acute febrile illnesses [3]. Diagnostic methods vary according to the stage of infection and include reverse transcription polymerase chain reaction (RT-PCR), non-structural protein 1 (NS1) antigen detection, and serological assays [6,7,8]. Among these, enzyme-linked immunosorbent assay (ELISA) remains widely used because of its relatively high sensitivity, affordability, and suitability for routine diagnostic laboratories and epidemiological investigations [8]. Detection of dengue-specific immunoglobulin M (IgM) antibodies provides evidence of recent infection, although interpretation requires caution because antibody responses depend on the timing of specimen collection and may be influenced by cross-reactivity with other circulating flaviviruses [9].

Although the immunopathogenesis of dengue, including cytokine-mediated inflammation and antibody-dependent enhancement, is well recognized [10,11,12,13,14], these mechanisms were not directly investigated in the present study. Consequently, they are considered only where relevant to the interpretation of serological findings.

Sero-epidemiological studies provide useful preliminary information on recent dengue virus exposure and can assist in strengthening surveillance programs, particularly in endemic regions where laboratory data remain limited [6,15]. Pilot studies are especially valuable for assessing methodological feasibility and generating baseline data that can guide larger prospective investigations [16,17].

Therefore, the aim of this retrospective pilot study was to evaluate anti-dengue virus IgM serological reactivity in anonymized serum samples from Trinidad and Tobago using a commercial ELISA. Specifically, the study sought to determine the proportion of positive, borderline, and negative IgM results and to interpret these findings within the context of dengue surveillance in an endemic Caribbean setting. Given the retrospective laboratory-based design, the findings are intended to provide preliminary evidence to support future studies incorporating molecular diagnostics, paired serology, and larger, population-based investigations.

## 2. Objectives of the Study

To evaluate serological and immunological markers associated with recent dengue virus infection through the detection of anti-dengue virus immunoglobulin M (IgM) antibodies in serum samples obtained from Trinidad and Tobago using an enzyme-linked immunosorbent assay (ELISA) [17].

## 3. Materials and Methods

### 3.1. Study Design

This study was conducted as a retrospective observational laboratory-based pilot investigation designed to evaluate serological markers associated with dengue virus infection. Pilot studies are valuable methodological tools because they allow assessment of feasibility, refinement of laboratory procedures, and generation of preliminary data that may inform larger epidemiological investigations [17]. The study focused on the laboratory detection of anti-dengue virus IgM antibodies and the interpretation of serological patterns associated with recent infection.

### 3.2. Study Setting

Data were obtained from original laboratory analyses performed in a clinical diagnostic and immunology laboratory in Trinidad and Tobago (UWI Immunology Laboratory) equipped for routine serological testing. Laboratory facilities included ELISA instrumentation, centrifugation equipment, refrigerated storage systems, and standard biosafety infrastructure appropriate for handling human serum specimens [8].

Trinidad and Tobago is located in the southern Caribbean and remains an endemic region for dengue virus transmission. Climatic conditions, seasonal rainfall patterns, and the widespread presence of *Aedes aegypti* mosquitoes contribute to the continued circulation of dengue virus throughout the country [2,3].

### 3.3. Study Population and Sample Selection

The study included serum samples submitted for dengue serological testing between 1 September 2025, and 28 February 2026.

Serum samples submitted for routine laboratory testing were included in this pilot laboratory study. Samples were processed in four independent analytical batches, comprising 31 samples in Batch 1, 29 samples in Batch 2, 53 samples in Batch 3, and 48 samples in Batch 4, giving a total of 161 specimens. Inclusion criteria consisted of adequate serum volume, complete laboratory documentation, and valid ELISA results. Duplicate submissions and specimens with incomplete laboratory data were excluded. All samples were anonymized before analysis, and no patient-identifying information was available to the investigators.

### 3.4. Sample Size

A total of 161 serum samples were included in the study. This sample size was considered appropriate for a pilot investigation intended to generate preliminary sero-epidemiological data rather than to provide definitive population estimates. Pilot studies are not typically powered for hypothesis testing but are useful for identifying trends, evaluating methodologies, and informing future study design [17].

### 3.5. Serological Analysis

Serological testing was performed using the Euroimmun anti-dengue virus ELISA (IgM) assay according to the manufacturer’s instructions. The assay is a semi-quantitative enzyme-linked immunosorbent assay designed for the detection of human IgM antibodies directed against dengue virus antigens.

The ELISA method is widely used in dengue surveillance because of its high sensitivity, relatively low cost, and suitability for large-scale epidemiological investigations [13,14]. Following infection, dengue-specific IgM antibodies generally become detectable approximately 3–5 days after symptom onset and may persist for several weeks, making IgM an important marker of recent infection [3,12].

Briefly, diluted serum samples were incubated in antigen-coated microplate wells. Bound antibodies were detected using enzyme-labeled anti-human IgM conjugates followed by chromogenic substrate development. Optical density values were measured spectrophotometrically at 450 nm and interpreted according to manufacturer-recommended criteria.

### 3.6. Interpretation of ELISA Results

Results were classified according to the manufacturer’s instructions as follows:

- Negative: ratio < 0.80.
- Borderline (equivocal): ratio ≥ 0.80 to <1.10.
- Positive: ratio ≥ 1.10.

Positive results were interpreted as evidence of recent or acute dengue virus infection. Borderline results were considered potentially indicative of early seroconversion, declining antibody levels, secondary dengue infection, or cross-reactive flavivirus antibodies and therefore required cautious interpretation [14,15].

### 3.7. Data Collection and Management

Each sample was assigned a unique study identification code to ensure anonymity. Data quality checks were performed before analysis to verify completeness, consistency, and accuracy.

The variables analyzed included:

- Serological classification (positive, borderline, or negative).
- Total number of samples within each category.
- Percentage distribution of serological findings.
- Batch-to-batch reproducibility.

### 3.8. Statistical Analysis

Data analysis was performed using descriptive statistical methods. Frequencies and percentages were calculated for positive, borderline, and negative serological results.

Seroprevalence was calculated using the following formula:

$$\mathrm{Seroprevalence}\;(\%)=\frac{\mathrm{Number}\;\mathrm{of}\;\mathrm{positive}\;\mathrm{samples}}{\mathrm{Total}\;\mathrm{number}\;\mathrm{of}\;\mathrm{samples}\;\mathrm{analyzed}}\times 100$$

Sero-prevalence was calculated using three predefined analytical strategies: (i) conservative (borderline results classified as negative); (ii) exclusion (borderline results omitted from analysis); and (iii) liberal (borderline results classified as positive). Exact 95% confidence intervals (CIs) were calculated using the Clopper–Pearson method. Batch-to-batch reproducibility was evaluated using Cochran’s Q heterogeneity test.

Statistical heterogeneity was quantified using the I^2^ statistic, with *p* < 0.05 considered statistically significant. Calculations were performed in R (R Foundation for Statistical Computing, Vienna, Austria).

### 3.9. Ethical Considerations

Ethical approval for the study was obtained from the relevant Campus Ethical Committee of the University of the West Indies at St. Augustine in Trinidad and Tobago prior to commencement of the investigation. The study was conducted in accordance with the ethical principles outlined in the Declaration of Helsinki [18].

## 4. Results

### Serological Distribution of Anti-Dengue Virus IgM Antibodies

A total of 161 serum samples were analyzed for the presence of anti-dengue virus IgM antibodies using a commercial ELISA. Samples were classified according to manufacturer-defined criteria as positive, borderline, or negative. Overall, 20 samples (12.4%) were positive, 29 samples (18.0%) were classified as borderline, and 112 samples (69.6%) were negative for anti-dengue virus IgM antibodies. These findings indicate evidence of recent dengue virus exposure within a proportion of the study population while demonstrating that most analyzed samples lacked serological evidence of recent infection.

The distribution shown in Table 1 demonstrates that negative results represented the largest category, accounting for nearly seventy percent of analyzed samples. Positive IgM results accounted for approximately one-eighth of the study population, while borderline results represented a substantial proportion of samples.

Overall, the study identified 20 positive samples, corresponding to an anti-dengue virus IgM seroprevalence of 12.4%. Borderline results accounted for 18.0% of analyzed samples, while 69.6% were negative. The relatively large borderline group may reflect early infection, declining antibody levels following recent infection, secondary dengue infection, or serological cross-reactivity with other circulating flaviviruses.

Under the conservative analytical approach, the estimated dengue IgM seroprevalence was 12.4% (20/161; 95% CI: 7.7–18.6%). Excluding borderline samples increased the estimated seroprevalence to 15.2% (20/132; 95% CI: 9.6–22.6%), whereas classifying all borderline samples as positive yielded a prevalence of 30.4% (49/161; 95% CI: 23.4–38.3%).

Batch-specific seroprevalence estimates ranged from 10.3% to 15.1%, with no significant heterogeneity among analytical batches (Q = 0.41, *p* = 0.94; I^2^ = 0%), demonstrating reproducibility across laboratory runs.

Published validation studies of the EUROIMMUN anti-dengue virus ELISA (IgM) have reported a sensitivity of 90.0% (95% CI: 84.5–93.9%) and a specificity of 98.0% (95% CI: 95.4–99.3%), supporting the use of the conservative analytical strategy adopted in this study [19].

## 5. Discussion

### 5.1. Principal Findings

This retrospective pilot study evaluated anti-dengue virus IgM seroprevalence among 161 serum samples collected in Trinidad and Tobago. The overall IgM sero-positivity rate of 12.4% indicates evidence of recent dengue virus exposure within the analyzed population, while an additional 18.0% of samples demonstrated borderline serological reactivity. These findings support the continued endemic circulation of dengue virus in Trinidad and Tobago and are broadly consistent with the epidemiological profile of dengue throughout the Caribbean region, where recurrent outbreaks and seasonal transmission remain important public health concerns [1,2,3].

Although the observed seroprevalence appears modest, its significance should not be underestimated. The samples analyzed were not collected during a recognized epidemic surge but rather represented routine laboratory submissions. Consequently, the presence of detectable IgM antibodies may reflect ongoing background transmission rather than outbreak-associated clustering. This observation reinforces the concept that dengue transmission in Trinidad and Tobago may occur continuously throughout the year, with amplification during favorable climatic periods.

### 5.2. Interpretation of the Borderline Serological Population

One of the most interesting findings of this study was the relatively high proportion of borderline ELISA results. Nearly one-fifth of analyzed samples demonstrated equivocal reactivity, raising important immunological and diagnostic questions.

Several explanations may account for this finding probabilistically. First, some individuals may have been tested during the early stages of infection before complete IgM maturation had occurred. Second, declining antibody concentrations during the convalescent phase may produce borderline results despite recent infection. Third, secondary dengue infections may exhibit altered antibody kinetics characterized by rapid IgG responses and relatively attenuated IgM production [4].

However, a more critical interpretation must acknowledge the possibility of flavivirus cross-reactivity. ELISA-based assays are known to exhibit reduced specificity in regions where multiple flaviviruses circulate. Antibodies directed against Zika virus, Yellow Fever virus, West Nile virus, and other related flaviviruses may generate low-level reactivity that is difficult to distinguish from true dengue-specific responses [5]. Without confirmatory neutralization assays or molecular testing, the exact nature of these borderline reactions remains uncertain.

This limitation highlights an important challenge faced by diagnostic laboratories throughout tropical regions and emphasizes the need for integrated diagnostic strategies that combine serological, molecular, and clinical information.

### 5.3. Critical Evaluation of the ELISA Methodology

The present study relied exclusively on IgM ELISA testing. While ELISA remains one of the most practical and widely used methods for dengue surveillance, several methodological limitations must be considered when interpreting the findings.

The principal strength of ELISA lies in its accessibility, affordability, and suitability for large-scale screening programs [6]. In resource-limited settings, ELISA provides a valuable tool for estimating seroprevalence and monitoring transmission trends. However, ELISA is fundamentally an indirect marker of infection because it measures host immune responses rather than direct viral presence.

Consequently, the timing of sample collection becomes critically important. Patients tested very early in infection may not yet have developed detectable IgM antibodies, resulting in false-negative findings. Conversely, IgM antibodies may persist for weeks or months following infection, potentially complicating interpretation of recent exposure [2].

A major criticism of the current study is the absence of NS1 antigen detection and RT-PCR confirmation. Molecular techniques provide significantly greater diagnostic specificity during the acute phase of infection and would have allowed confirmation of active dengue infection among seropositive individuals. The lack of molecular confirmation therefore limits the diagnostic certainty of the reported seroprevalence.

Future studies should incorporate a multimodal diagnostic approach including NS1 antigen testing, RT-PCR, IgM ELISA, and IgG serology to improve diagnostic accuracy and provide a more comprehensive assessment of dengue epidemiology.

### 5.4. Epidemiological Implications

The epidemiological findings are consistent with established patterns of dengue transmission in Trinidad and Tobago and throughout the Caribbean. Seasonal peaks observed during the rainy months likely reflect increased mosquito breeding opportunities, greater vector density, and enhanced transmission efficiency [10].

The epidemiological figures included in this study demonstrate fluctuating annual transmission patterns, with apparent outbreak peaks occurring in selected years. These observations support previous reports indicating that dengue activity in the Caribbean is influenced by climatic variability, vector ecology, population immunity, and serotype circulation [11].

However, caution must be exercised when interpreting these observations. The present study was not designed as a population-based surveillance investigation and therefore cannot accurately estimate national incidence or prevalence rates. The analyzed samples represent a laboratory population rather than a representative sample of the broader Trinidad and Tobago population. This limitation constitutes one of the most important methodological criticisms of the study and restricts the generalizability of the findings.

### 5.5. Dengue IgM Seroprevalence Varies Widely Across the Globe

Dengue IgM seroprevalence varies widely across the globe, reflecting differences in transmission intensity, population immunity, and surveillance capacity. A recent systematic review of global dengue prevalence reported that IgM seropositivity averages around 15.6% worldwide, though individual studies show substantial variation depending on outbreak timing, local vector density, and diagnostic methods. Regions with intense transmission—particularly South-East Asia and parts of the Americas—tend to report higher IgM positivity due to frequent recent infections. These findings highlight the ongoing burden of acute dengue and the importance of timely detection of IgM antibodies, which indicate recent viral exposure [20].

Global analyses also show that dengue seroprevalence patterns differ across age groups and geographic settings, with IgM levels rising during active transmission seasons and declining as outbreaks subside. Large-scale serosurveys demonstrate significant heterogeneity in dengue force of infection, driven by urbanization, climate, and vector ecology. Age-stratified seroprevalence studies further reveal that populations in hyperendemic regions experience repeated infections, contributing to fluctuating IgM levels over time. Together, these data underscore the need for strengthened surveillance systems and standardized diagnostic approaches to accurately track recent dengue infections worldwide as shown in cited research [21,22]. Table 2 shows global dengue IgM seroprevalence: multi-region comparative summary.

### 5.6. Critical Analysis of IgM Seroprevalence in Trinidad Compared with Global Studies

Our pilot study’s 12.4% dengue IgM seroprevalence in Trinidad represents a meaningful indicator of recent viral transmission in a Caribbean setting where dengue is endemic but often under-documented. Compared with global MDPI-indexed studies, in Trinidad the IgM level is notably higher than the 5.9% pooled IgM seroprevalence reported in Ghana [23], suggesting that Trinidad experiences a more active force of infection than many West African settings. It also aligns closely with the upper range of IgM positivity (2–12%) reported in the Thailand–Laos seroconversion study [25], indicating that Trinidad’s transmission intensity is comparable to Southeast Asian regions during periods of heightened circulation. This positions Trinidad as a moderate-to-high transmission environment, consistent with the island’s climate, vector ecology, and year-round *Aedes aegypti* presence.

When compared with the 15.5% IgM seroprevalence reported among febrile patients in Dhaka, Bangladesh [26], Trinidad’s pilot study seroprevalence falls slightly lower, suggesting that while dengue transmission is active, it may not reach the hyperendemic levels observed in densely populated South Asian urban centers. Importantly, our result is substantially lower than the 34.2% pooled IgM seroprevalence reported in the SAARC meta-analysis [24], reinforcing that Trinidad’s epidemiological profile reflects consistent but not strong transmission. Overall, the 12.4% IgM finding is epidemiologically coherent: it signals ongoing recent infections, aligns with patterns seen in other tropical regions, and underscores the need for strengthened surveillance, especially given the Caribbean’s vulnerability to climate-driven vector expansion.

### 5.7. Comparison of IgM Seroprevalence Reported in Pilot Studies

Malaysia rural pilot study—This community-based pilot study in Southern Malaysia found 11.2% IgM positivity, indicating recent dengue infection among healthy adults. This level reflects active but not extreme transmission in rural Southeast Asia [27].

Trinidad ELISA pilot study—A retrospective ELISA-based pilot study in Trinidad detected 12.4% IgM positivity, slightly higher than Malaysia and consistent with ongoing dengue circulation in the Caribbean. Borderline IgM reactivity (18%) suggests early seroconversion or secondary infections [28].

VIDAS multicenter diagnostic study—Although not a seroprevalence survey, this multicenter evaluation across dengue-endemic regions reported moderate IgM assay agreement, supporting the reliability of IgM detection in pilot-scale studies. These findings help contextualize IgM performance across diverse settings. The VIDAS multicenter diagnostic study was conducted across multiple international dengue-endemic regions, specifically in Asia, Latin America, and Africa. This is clearly stated in the multicenter performance evaluation published in *Diagnostics (MDPI)*, which reports that 1296 patients were enrolled from these regions [29].

In summary, across pilot studies, IgM seroprevalence typically ranges from ~10 to 13%, placing the Trinidad value (12.4%) squarely within the expected band for regions with active but non-hyperendemic transmission. Malaysia’s 11.2% suggests similar recent-infection dynamics in rural Southeast Asia, while Trinidad’s slightly higher rate aligns with Caribbean seasonal transmission patterns. The VIDAS multicenter study reinforces that IgM ELISA detection is a dependable marker of recent infection across endemic regions [29], supporting the validity of the Trinidad pilot study findings. Table 3 depicts the integrated mechanisms explaining borderline dengue IgM results and elevated IgM seroprevalence.

Together, these mechanisms explain why 29 of 161 samples (≈18%) produced borderline IgM results and why IgM seroprevalence was elevated. The convergence of structural epitope conservation, immune memory, diagnostic antigen design, regional arbovirus circulation, travel-related exposure, and cytokine-driven polyclonal activation creates a strong biological basis for flavivirus IgM cross-reactivity. Table 4 provides updated evidence supporting flavivirus cross-reactivity relevant to IgM ELISA interpretation in Trinidad and Tobago.

In summary, these updated studies (2024–2026) collectively demonstrate that cross-reactivity is a consistent global challenge in flavivirus serology. They directly support the interpretation that the 29 borderline IgM results (18.01%) in Trinidad’s ELISA pilot study are biologically and diagnostically plausible due to: shared flavivirus epitopes, diverse exposure histories, co-circulating arboviruses, and ELISA antigen design.

### 5.8. Strengths of the Study

Despite its limitations, this investigation represents one of the few laboratory-based serological evaluations of dengue infection conducted in Trinidad and Tobago in recent years. Local data remain limited, and studies focusing on Caribbean populations are underrepresented in the scientific literature. Second, the study utilized standardized ELISA methodology and predefined interpretation criteria, improving reproducibility and facilitating comparison with other investigations.

### 5.9. Study Limitations and Critical Appraisal

Several important limitations warrant careful consideration. The retrospective design inherently restricts control over data quality, sample selection, and clinical information. The sample size was relatively small and derived from a single laboratory source at Eric Williams Medical Science Complex, University of the West Indies Immunology Laboratory.

The absence of demographic variables in the study represents another limitation. Perhaps the most significant limitation is the lack of molecular confirmation. Finally, potential flavivirus cross-reactivity remains an unresolved concern. Given the epidemiological context of the Caribbean, some positive or borderline findings may not necessarily represent true dengue infection.

### 5.10. Future Directions

Future research should focus on prospective multicenter studies involving larger sample sizes and integration of molecular diagnostics. Studies would also help clarify the role of antibody-dependent enhancement (ADE) and its contribution to severe disease.

### 5.11. Overall Interpretation

Overall, this pilot study provides preliminary evidence supporting continued dengue virus circulation in Trinidad and Tobago. However, the findings should be interpreted cautiously because of the limitations of this study and they are best viewed as a hypothesis-generating exploratory investigation.

## 6. Conclusions

This retrospective pilot study showed an anti-dengue IgM seroprevalence of 12.4% among analyzed serum samples from Trinidad and Tobago, supporting continued endemic dengue virus circulation within the country. The presence of a borderline serological population further highlights the complexity of dengue immune responses and the challenges associated with serological interpretation in flavivirus-endemic regions. ELISA-based surveillance remains a useful diagnostic approach, particularly in resource-limited settings like Trinidad and Tobago. Future investigations incorporating molecular diagnostics, paired serology and larger multicenter cohorts are required to better define dengue epidemiology in the country and immuno-pathogenesis in the wider Caribbean region.

The estimated seroprevalence of dengue IgM in this pilot study was highly dependent on the analytical treatment of borderline ELISA results. Conservative classification provided the most cautious and reproducible estimate of recent dengue infection, while liberal interpretation substantially increased the apparent disease burden. These findings emphasize the importance of transparent reporting and standardized interpretation of equivocal serological results to improve the accuracy and comparability of dengue surveillance data, and support evidence-based public health decision-making.

Commercial ELISAs frequently incorporate whole-virus antigens or recombinant E protein containing conserved epitopes, enhancing sensitivity but also cross-reactivity. Epidemiological conditions in Trinidad—where multiple arboviruses circulate—mirror settings in Bangladesh and Croatia, where overlapping IgM responses to dengue, chikungunya, and Zika have been documented. Additionally, imported cases introduce diverse exposure histories, further broadening the serological profile. Immune dysregulation driven by cytokine activation during acute infection also promotes polyclonal B cell responses, amplifying cross-reactive IgM production.

Together, these immunological, diagnostic, and epidemiological factors provide a robust explanation for the elevated IgM seroprevalence and borderline reactivity observed in this study. The results underscore the need for cautious interpretation of IgM ELISA results, particularly in regions with co-circulating arboviruses or diverse travel histories. Future diagnostic strategies in Trinidad may benefit from incorporating confirmatory assays, neutralization tests, or epitope-refined antigen designs to improve specificity. Ultimately, understanding the drivers of IgM cross-reactivity is essential for accurate dengue surveillance, outbreak detection, and clinical decision-making.

## Figures and Tables

**Table 1:** Summary of ELISA IgM serological results.

ELISA Batch	Total Samples	Positive	Borderline	Negative
Batch 1	31	5	1	25
Batch 2	29	2	2	25
Batch 3	53	7	4	42
Batch 4	48	6	18	24
Total	161	20	29	112

**Table 2:** Global dengue IgM seroprevalence: multi-region comparative summary.

Region/Study	IgM Seroprevalence	Population/Setting	Reference
Ghana review	5.9%	Febrile patients & blood donors (systematic review)	[23]
SAARC territory meta-analysis	34.2%	Suspected dengue cases across South Asia	[24]
Thailand–Laos seroconversion study	2–12% (IgM positivity varies by season & setting)	Urban & rural community serosurveys	[25]
Bangladesh febrile-patient study	15.5%	Febrile patients in Dhaka hospitals	[26]

**Table 3:** Integrated mechanisms explaining borderline dengue IgM results and elevated IgM seroprevalence.

Mechanism	Explanation	Supporting References
**1. Conserved Flavivirus E Epitopes Drive Broad IgM Binding**	Early IgM responses have low affinity but high avidity, enabling binding to conserved epitopes shared across DENV, ZIKV, WNV, and JEV. This broad reactivity increases borderline ELISA signals.	[30,31]
**2. Prior Exposure or Vaccination Creates Cross-Reactive Memory Pools**	Previous flavivirus infections or vaccinations generate memory B cells that recognize conserved epitopes. Upon new DENV infection, these cross-reactive memory cells are reactivated, producing IgM that binds multiple flaviviruses.	[32,33]
**3. ELISA Antigens Contain Shared Epitopes**	Commercial IgM ELISAs often use whole-virus antigens or recombinant E protein containing conserved epitopes. This increases sensitivity but also cross-reactivity, producing borderline results.	[34,35]
**4. Co-circulation of Multiple Arboviruses**	In regions where DENV, ZIKV, CHIKV, and WNV co-circulate, individuals may have overlapping antibody profiles. This increases IgM cross-reactivity and borderline ELISA outcomes.	[36,37]
**5. Imported Cases Increase Serological Diversity**	Travel-related infections introduce individuals with diverse flavivirus exposure histories, increasing heterologous IgM responses and borderline serological patterns.	[38,39,40]
**6. Cytokine-Driven Immune Dysregulation Broadens Antibody Responses**	Strong cytokine activation (TNF-α, IL-6, IL-10, IFN-γ) during DENV infection promotes polyclonal B-cell activation, broadening IgM repertoires and increasing cross-reactivity.	[10,11,12,13,14]

**Table 4:** Updated evidence (2024–2026) supporting flavivirus cross-reactivity relevant to IgM ELISA interpretation in Trinidad and Tobago.

Study/Region	Key Finding	Relevance to IgM Cross-Reactivity & Borderline Results	Reference
**Imported dengue cases in Europe (Italy)**	Frequent serological ambiguity due to prior flavivirus exposure or vaccination.	Demonstrates how diverse exposure histories (travel, vaccination) increase heterologous IgM responses, mirroring Trinidad’s mixed-exposure population.	[38]
**ZIKV serological assay comparison (Global blinded panels)**	Strong cross-reactivity between ZIKV and DENV IgM/IgG across multiple commercial assays.	Confirms that ELISA platforms commonly used worldwide—including those similar to Trinidad’s pilot—produce cross-reactive IgM signals.	[34]
**Re-emergence of dengue in Croatia**	Diagnostic overlap between DENV and other arboviruses complicates outbreak confirmation.	Shows that even in non-endemic regions, flavivirus IgM cross-reactivity is substantial, supporting the plausibility of borderline IgM results in Trinidad.	[37]
**Bangladesh febrile patient seroprevalence**	Overlapping IgM positivity for dengue, chikungunya, and Zika.	Demonstrates real-world IgM cross-reactivity in co-circulating arbovirus settings similar to Trinidad’s epidemiological profile.	[36]
**Multiepitope ELISA development (ASFV model)**	ELISAs using multiple conserved epitopes increase sensitivity but also cross-reactivity.	Provides mechanistic evidence that multiepitope antigen design—similar to many flavivirus ELISAs—can elevate borderline IgM rates.	[35]

## Data Availability

The dataset supporting the findings of this study is included in the manuscript and its referenced sources, ensuring comprehensive access to the relevant data for further examination and analysis.
